# Universal Latent Representation in Finite Ring Continuum

**DOI:** 10.3390/e28010040

**Published:** 2025-12-28

**Authors:** Yosef Akhtman

**Affiliations:** 1 Gamma Earth Sàrl, 1162 St-Prex, Switzerland; ya@gamma.earth; Tel.: +41-75-4179129; 2 Faculty of Space Technologies, AGH University of Krakow, 30-059 Krakow, Poland

**Keywords:** finite fields, relational finitude, latent structure, minimal sufficiency, modular arithmetic, innovation–consolidation, exploration–exploitation, foundational representations, complexity, cross-modal alignment

## Abstract

We propose a unified mathematical framework showing that the representational universality of modern foundational models arises from a shared finite latent domain. Building on the Finite Ring Continuum (FRC) framework, we model all modalities as epistemic projections of a common latent set $Z\subset U_{t}$, where $U_{t}$ is a symmetry-complete finite-field shell. Using the uniqueness of minimal adequate representations, we prove the Universal Subspace Theorem, establishing that independently trained embeddings coincide, up to bijection, as coordinate charts on the same latent structure. This result explains cross-modal alignment, transferability, and semantic coherence as consequences of finite relational geometry rather than architectural similarity. The framework links representation learning, sufficiency theory, and FRC algebra, providing a principled foundation for universal latent structure in multimodal models.

## 1. Introduction

Foundational models—large-scale deep learning systems trained on diverse modalities such as natural language, images, audio, and geospatial data—have demonstrated an unexpected degree of representational universality. Despite differences in architecture, training objectives, and input domains, the internal embeddings produced by these models exhibit striking structural similarities, including cross-modal alignment, semantic coherence, and shared latent geometry [1,2,3,4]. These empirical regularities suggest the presence of a deeper unifying principle underlying representation formation across modalities.

Two major theoretical traditions attempt to explain this phenomenon. The first, rooted in classical statistical learning theory, views representations through the lens of sufficient statistics [5,6,7,8]. From this perspective, a learned embedding is successful when it preserves all task-relevant information about an underlying latent variable. The second tradition focuses on the geometric and algebraic structure of deep learning systems, emphasizing invariances, symmetry, compression, and latent manifold organization [9,10,11,12,13]. Both viewpoints capture important aspects of representation learning, yet neither fully explains why multimodal embeddings appear to inhabit compatible latent spaces, or why transfer between modalities is unexpectedly effective.

In this work, we propose a unifying explanation based on the Finite Ring Continuum (FRC) [14,15,16]. The FRC is a finite, relational, and symmetry-complete algebraic framework that models arithmetic structure through a hierarchy of relational symmetry spaces called shells:

$$U_{t}\;\subset \;U_{t+1}\;\subset \;U_{t+2}\;\subset \;\cdots$$

indexed by a discrete “shell radius”. Within this architecture, arithmetic symmetries induce a combinatorial geometry that supports both Euclidean and Lorentzian structures, while the shell hierarchy encodes increasing algebraic and geometric complexity. The FRC therefore provides a natural candidate for a universal latent domain shared across modalities.

Our main contribution is to connect the FRC framework with classical sufficiency theory and multi-view latent variable modelling. Building on the assumption that each modality observes a noisy projection of a common latent domain Z, we show that foundational embeddings trained on different modalities recover injective transformations of the same latent set. A key result from statistics asserts that minimal sufficient representations are unique up to bijection. We combine this with the finite-field geometry of the FRC to obtain the *Universal Subspace Theorem* (Theorem 1), which states that all foundational embeddings correspond to coordinate charts on a single latent set $Z\subset F_{p}$, embedded into a shared arithmetic shell.

This theorem provides a structural explanation for cross-modal representational alignment: multimodal embeddings agree because they are necessarily different coordinate representations of the same latent world variable. In the canonical parametrization, they coincide exactly. This insight unifies notions of sufficiency, multimodal learning, and deep representation geometry within a single algebraic framework.

Beyond the main theoretical result, we explore interpretive connections between network depth and arithmetic shell hierarchy, the role of nonlinearity in expressive expansion, and the conceptual relationship between latent-field reconstruction and modern self-supervised learning objectives. These connections suggest that foundational models implicitly operate on finite-field latent manifolds whose structure reflects deep algebraic symmetry.

Importantly, this work does not aim to derive deep learning from SGD dynamics, but to demonstrate that the representational geometry emerging in foundational models is consistent with and predicted by a finite relational ontology. Overall, this work contributes a principled theoretical foundation for understanding why foundational models generalize across modalities, why their embeddings exhibit universal alignment, and how discrete arithmetic structure may underlie the geometry of learned representations.

A growing body of prior work in machine learning provides empirical and theoretical support for our central premise of Universal Latent Representation. For example, Zhang et al. [17] demonstrated that over-parameterized neural networks can perfectly interpolate arbitrary labels, thereby decoupling memorization capacity from generalization and implying that generalization must be driven by structure inherent to the data themselves. Power et al. [18] subsequently provided a controlled empirical setting in which models trained on purely relational, attribute-free symbols exhibit abrupt transitions from memorization to perfect generalization, accompanied by the emergence of clear algebraic geometry in the learned embeddings, a phenomenon consistent with the resolution of a latent relational domain.

Wei et al. [19] documented sharp, non-smooth phase transitions in the capabilities of large language models as scale increases, a phenomenology that aligns with the interpretation of capability emergence as latent-structure accessibility rather than gradual statistical improvement. Hernandez et al. [20] showed that transfer performance only scales when tasks share an underlying structure, indicating that increased computation alone does not generate new representational scope in the absence of latent overlap. Finally, Burns et al. [21] demonstrated that large language models encode recoverable latent knowledge not explicitly supervised during training, reinforcing the distinction between latent representational structure and the training signals used to elicit it.

Taken together, these works support the ULR view that learned representations act as coordinate realizations of an underlying latent structure determined primarily by the data domain. Notably, and perhaps unfortunately, the results of this explanatory nature have become comparatively rare following the emergence of large-scale models such as ChatGPT-4, as much of the recent academic focus has shifted from explainability and structural analysis toward scalability, performance, and deployment-oriented concerns.

Finally, it should be noted that the results of this paper are formulated purely at the representation level and rely only on the finiteness of the latent domain Z. No algebraic or geometric properties of the ambient shell $U_{t}$ are required for the presented proofs. The necessity and uniqueness of the Finite Ring Continuum as the host structure for such finite latent domains follow from independent ontological arguments based on finitude and timefulness, further developed in our follow-up work [22].

**Notation.** Throughout, calligraphic symbols (e.g., $Z,X_{m},U_{t}$) denote sets, while lowercase symbols denote elements. Maps are written explicitly with their domain and codomain (e.g., $E_{m}:X_{m}\to W_{m}$), and images are identified with subsets only when this causes no ambiguity. Informal phrases such as “factors through” are used in the standard set-theoretic sense of explicit map composition. $\varphi_{m}$ denotes the latent coordinate map, while $\psi_{m}$ is reserved for the canonical representation map of Definition 2.

## 2. Background

This section reviews the mathematical foundations on which our framework is built. We summarize (i) the relevant aspects of the Finite Ring Continuum (FRC), (ii) standard principles of representation learning, and (iii) the multi-view latent-variable formalism used to articulate the connection between embeddings and latent arithmetic structure.

### 2.1. Finite Ring Continuum: Algebraic and Geometric Preliminaries

The Finite Ring Continuum (FRC), as developed in [14,15,16], constructs a hierarchy of discrete arithmetic universes based on *symmetry-complete prime fields*

$$F_{p},\;p=4t+1,$$

the multiplicative groups of which contain the structural set {1,i,−1,−i} satisfying $i^{2}=-1$. This guarantees the existence of 4-element rotational orbits and a meaningful geometric interpretation of the arithmetic operations.

Within each shell of radius *t*, the arithmetic symmetries generated by translations $T_{a}\left(x\right)=x+a$, multiplications $S_{m}\left(x\right)=mx$, and power maps $P_{\varepsilon }\left(x\right)=x^{\varepsilon }$ act on $F_{p}$ to produce a combinatorial 2-sphere $S_{p}$ embedded in a symbolic (1,3)-dimensional space $U={⋃}_{t}U_{t}$; see [14] for details. These shells support two qualitatively distinct transformation classes:

**Consolidation steps:**  reversible, symmetry-preserving operations internal to $F_{p}$ (e.g., translation, scaling);
**Innovation steps:**  algebraic extensions such as $F_{p}↪F_{p^{2}}$, which introduce new square classes and enable a Lorentzian structure [15].

In this work, a fixed shell $U_{t}$ will serve as the ambient space for the latent domain Z in Section 3. Since $U_{t}$ is finite, any subset $Z\subseteq U_{t}$ is finite as well—an observation that will be relevant when embedding learned representations into a common shell.

It should also be noted that the restriction to cardinalities q≡1(mod4) is not number-theoretic in origin but follows from the ontological commitments of finitude and timefulness detailed in [22]. Specifically, finitude enforces a spatial involution R(x)=−x on the cyclic enumeration, while timefulness requires a second involution that cannot be reduced to spatial reversal. The coexistence of two distinct involutive symmetries therefore forces symmetry-complete shells, i.e., shells admitting an internal Euclidean conjugation already at the shell level.

Prime cardinalities p≡1(mod4) provide irreducible and maximally symmetric realizations of this structure and are used as convenient key-frame models in the present analysis. However, the Finite Ring Continuum does not require primality: composite cardinalities of the same form are equally admissible. For sufficiently large *q*, any local observer with a finite informational horizon cannot distinguish prime from composite structures; locally, composite shells are operationally indistinguishable from prime ones.

### 2.2. Representation Learning and Embedding Geometry

Deep representation learning systems construct feature maps by alternating affine transformations and nonlinear activation functions. For an input *x*, a typical embedding has the form

$$E\left(x\right)=L^{\left(n\right)}\sigma \left(L^{\left(n-1\right)}\sigma \left(\cdots \sigma \left(L^{\left(1\right)}x\right)\right)\right),$$

where the $L^{\left(k\right)}$ are linear operators and σ denotes a nonlinear activation. This alternating structure is essential for universal approximation and for the formation of expressive latent manifolds.

Empirically, large foundational models trained on different modalities (e.g., language, vision, audio, remote sensing) produce embeddings that exhibit unexpectedly coherent geometric relationships, including cross-modal alignment and shared semantic directions. The present work provides a formal explanation of this phenomenon by showing that all such embeddings can be interpreted as coordinate representations of a single latent structure embedded in an FRC shell.

### 2.3. Multi-View Latent Domain Models

We adopt a standard multi-view formalism in which observable data from each modality *m* arise as a transformation of a common latent domain Z:

(1) $$X_{m}=g_{m}\left(Z,\varepsilon_{m}\right),$$

where $\varepsilon_{m}$ denotes unresolved modality-specific degrees of freedom. We assume that all cross-modal relations are mediated exclusively by the latent domain Z, in the sense that for any m≠n there exists no additional structural coupling between $X_{m}$ and $X_{n}$ beyond their common dependence on Z. Equivalently, any shared relational content between modalities factors through Z alone.

An adequate representation of the latent domain Z based on observations $X_{m}$ is any map $E_{m}:X_{m}\to W_{m}$ for which there exists an injective map $\phi_{m}:Z\to W_{m}$ such that

$$E_{m}\left(X_{m}\right)=\phi_{m}\left(Z\right),$$

where the representation factors through the latent domain itself. An adequate representation is called *minimal* if $\phi_{m}$ is bijective onto its image, so that no further nontrivial quotient of $W_{m}$ preserves this factorization.

A classical structural result, traditionally formulated in the language of sufficient statistics [5,6], admits the following set-theoretic reformulation.

**Lemma** **1** (Uniqueness of Minimal Adequate Representations)**.** *If $E_{m}$ and $E_{m}^{\prime }$ are minimal adequate representations of Z based on $X_{m}$, with*

$$E_{m}\left(X_{m}\right)=\phi_{m}\left(Z\right),\;E_{m}^{\prime }\left(X_{m}\right)=\phi_{m}^{\prime }\left(Z\right),$$

*where $\phi_{m}:Z\to W_{m}$ and $\phi_{m}^{\prime }:Z\to W_{m}^{\prime }$ are bijections onto their images, then there exists a unique bijection*

$$\psi_{m}:W_{m}\to W_{m}^{\prime }$$

*such that*

$$E_{m}^{\prime }\left(X_{m}\right)=\psi_{m}\;\left(E_{m}\left(X_{m}\right)\right).$$

Lemma 1 implies that in the idealized regime of infinite data and model capacity, a learned embedding $E_{m}$ must coincide (up to a bijection) with an injective function of Z. Consequently, $E_{m}\left(X_{m}\right)$ ranges over a finite set $W_{m}=\phi_{m}\left(Z\right)\subset R^{d_{m}}$, where $\phi_{m}$ is injective on Z.

Since $Z\subset U_{t}$ and $W_{m}=\varphi_{m}\left(Z\right)$ with $|W_{m}\left|=|Z\right|$, there exists an injective embedding $W_{m}↪U_{t}$. In Section 4, we show that under this construction, all embeddings from all modalities embed into isomorphic images of the same latent set.

## 3. Theoretical Framework

In this section, we formalize the setting in which foundational embeddings are analyzed. Unlike classical statistical treatments, we do not assume infinite populations, continuous probability measures, or aleatoric randomness. Instead, we adopt the ontological perspective of the Finite Ring Continuum (FRC) [14,15,16], where all information is fundamentally finite, and all uncertainty arises from limited observer access rather than intrinsic stochasticity. This shift eliminates the need for classical conditional independence assumptions and replaces them with a finite, relational picture of latent structure.

### 3.1. The Ontological Necessity of the Latent Arithmetic Universe

We posit that the fundamental elements of the latent domain must be genuinely primitive: they admit no intrinsic attributes and are therefore mutually indistinguishable. This echoes the principle of indistinguishable quanta in fundamental physics, but here we take the idea to its logical completion. If primitives are attribute-free, they cannot participate in a linear order, as any such order would implicitly assign a distinguished “first” or “last” element or require an external coordinate background. The only permissible relational structure among indistinguishable primitives is a cycle—a closed relational orbit in which no element is privileged.

Combined with the logical necessity of finiteness [23], required to avoid paradoxes associated with infinite informational content, the relational cycle acquires an arithmetic interpretation: counting a finite set of indistinguishable elements necessarily yields a cyclic group, and the natural extension of this structure to a full arithmetic system is a finite ring, and in particular a finite field when symmetry completeness is required. Thus, the latent domain is not merely assumed to reside in a finite ring; rather, under the premises of primitivity, indistinguishability, and finiteness, it is forced to do so. In this sense, the Finite Ring Continuum is not an arbitrary modelling choice but the unique algebraic geometry compatible with a universe composed of distinct yet attribute-free primitives.

Following an even broader FRC ontology, the physical universe itself is modelled as a finite collection of attribute-free primitive elements. Such primitives cannot bear intrinsic labels, order, or internal attributes; hence, all structures must emerge relationally. As shown in [14,15], any finite set of identical primitives admits an emergent counting operation, which induces a cyclic arithmetic structure and leads naturally to a finite-field shell.

Let p=4t+1 be prime, and let $U_{t}$ denote the corresponding finite-field shell of radius *t*. The shell $U_{t}$ provides a homogeneous, attribute-free arithmetic universe capable of supporting emergent geometry and causal structures, as detailed in [15,16].

**Definition** **1** (Finite Relational Latent Domain)**.** *Let $Z\subset U_{t}$ be a finite set. We interpret Z as the complete relational domain internally reconstructed by a given model from the data—namely, a collection of manifestations of $U_{t}$—made available to it.*

For each modality *m* (e.g., text, image, audio, geospatial data), observations arise as deterministic projections of the finite relational domain Z. We model this using a map

(2) $$g_{m}:Z\to X_{m},$$

which reflects the observer’s finite horizon and modality-specific resolution.

Equation (2) does not represent a stochastic generative model. Rather:

(i) Any apparent randomness in $X_{m}$ arises from the observer’s epistemic limitations, not from intrinsic aleatoric processes.
(ii) No conditional independence assumptions among modalities are required. Different modalities may reveal overlapping or non-overlapping relational aspects of the same latent state.

This epistemic interpretation is consistent with the relational nature of FRC and avoids reliance on classical probability theory, whose infinite-population constructs are incompatible with the finite informational ontology of the universe.

### 3.2. Foundational Embeddings as Sufficient Representations

A foundational model for modality *m* produces a representation,

$$E_{m}:X_{m}\to R^{d_{m}}.$$

We interpret $E_{m}\left(x_{m}\right)$ for $x_{m}\in X_{m}$ as the information about the latent domain Z that the model is able to recover from the modality-specific projection $g_{m}$.

In classical statistics, a sufficient statistic preserves all information about an underlying state [5,6]. We invoke this notion only as a conceptual motivation. In the present finite, epistemic setting, no probabilistic structure is assumed, and the operative notion is purely set-theoretic.

**Definition** **2** (Adequate Representation)**.** *An embedding $E_{m}:X_{m}\to W_{m}$ is adequate if there exists a bijection*

$$\varphi_{m}:Z\to W_{m}$$

*such that*

$$E_{m}\circ g_{m}=\varphi_{m}.$$

Since $\varphi_{m}$ is injective and $E_{m}\circ g_{m}=\varphi_{m}$, adequacy implies that $g_{m}$ is injective on Z. Thus, Z is interpreted as the maximally resolved latent domain accessible to modality *m*, rather than as a pre-quotient space.

**Lemma** **2** (Uniqueness of minimal adequate representations up to bijection)**.** *Let $E_{1}:X\to W_{1}$ and $E_{2}:X\to W_{2}$ be minimal adequate representations factoring through the same finite domain Z in the above sense, i.e., $E_{i}=\phi_{i}\circ g$ with $\phi_{i}:Z↪W_{i}$ injective. Then there exists a bijection $\psi :W_{1}\to W_{2}$ such that $E_{2}=\psi \circ E_{1}$.*

**Proof.**  Since $\phi_{1}$ and $\phi_{2}$ are injective, their images $\phi_{1}\left(Z\right)\subseteq W_{1}$ and $\phi_{2}\left(Z\right)\subseteq W_{2}$ are in bijection via $\phi_{2}\circ \phi_{1}^{-1}$. Extend this bijection to $\psi :W_{1}\to W_{2}$ as follows: by minimality, $W_{i}=\phi_{i}\left(Z\right)$, otherwise collapsing $W_{i}\setminus \phi_{i}\left(Z\right)$ would yield a nontrivial quotient preserving adequacy. Thus, $\psi :=\phi_{2}\circ \phi_{1}^{-1}$ is a bijection $W_{1}\to W_{2}$ and $E_{2}=\psi \circ E_{1}$ follows from $E_{i}=\phi_{i}\circ g$. □

Furthermore, because $W_{m}$ is finite and $U_{t}$ is a finite shell, there exists an injective map,

$$\iota_{m}:W_{m}↪U_{t}.$$

We therefore define the composite map

$$\psi_{m}:=\iota_{m}\circ \phi_{m}:Z↪U_{t},$$

which embeds the recovered relational information into the common finite-field universe. The embeddings $\psi_{m}$ provide modality-specific coordinate systems on the same latent set Z within $U_{t}$. The Universal Subspace Theorem (Section 4) shows that these coordinate systems are bijectively related and can be unified into a canonical parametrization.

## 4. Universal Subspace Theorem

This section presents the central theoretical result of the paper. Under the multi-view assumptions and the finite-field latent structure developed in Section 3, we show that all foundational embeddings learned from different modalities give rise to isomorphic coordinate embeddings of the same latent set Z inside the arithmetic shell $U_{t}=F_{p}$. This establishes a rigorous basis for cross-modal representational alignment and shared semantic structure.

It is important to note that the contribution of this article is not the lemma itself, but the realization that the lemma becomes ontologically forced once the latent domain is finite, relational, and attribute-free. Alignment is not an accident of SGD; it is a structural consequence of finiteness.

Recall that for each modality *m* we have:

(i) a latent domain $Z\subset U_{t}$;
(ii) an observation map $g_{m}:Z\to X_{m}$;
(iii) a minimal adequate representation $E_{m}$ with $E_{m}\left(X_{m}\right)=\phi_{m}\left(Z\right)$, where $\phi_{m}:Z\to W_{m}$ is injective;
(iv) an injective map $\iota_{m}:W_{m}↪U_{t}$;
(v) the composite embedding $\psi_{m}=\iota_{m}\circ \phi_{m}:Z↪U_{t}$.

We use only the finiteness of Z and $W_{m}$, which follows from the finiteness of $U_{t}$, and the standard uniqueness of minimal sufficient statistics [5,6].

**Theorem** **1** (Universal Subspace Theorem)**.** *Under Definitions 1 and 2, as well as assuming that each foundational embedding $E_{m}$ is minimal adequate with respect to the finite domain Z, the following statements hold:*

*(i)*  *Each composite map*
  $$\psi_{m}=\iota_{m}\circ \phi_{m}:Z↪U_{t}$$
  *is injective.*
*(ii)*  *For any two modalities m,n, the images $\psi_{m}\left(Z\right)$ and $\psi_{n}\left(Z\right)$ are isomorphic as sets. More precisely, the map*
  $$\Psi_{m\to n}:=\psi_{n}\circ \psi_{m}^{-1}:\psi_{m}\left(Z\right)\to \psi_{n}\left(Z\right)$$
  *is a well-defined bijection.*
*(iii)*  *There exists a canonical choice of embeddings $\iota_{m}$ for which all $\psi_{m}$ coincide:*
  $$\psi_{m}=\psi_{n}=:\psi \;\mathrm{for}\;\mathrm{all}\;m,n.$$
  *In this canonical parametrization,*
  $$\psi \left(Z\right)=\psi_{m}\left(Z\right)=\psi_{n}\left(Z\right)\subset U_{t}$$
  *for all modalities.*

*Therefore, all foundational embeddings factor through isomorphic images of the same latent set Z inside the shell $U_{t}$:*

$$E_{m}\left(X_{m}\right)\;\overset{\phi_{m}}{⟷}\;Z\;\overset{\psi }{↪}\;U_{t}.$$

**Remark** **1.** *Theorem 1 is intentionally formulated without additional structure: its content is that alignment is forced by finiteness and minimal adequacy alone, prior to the introduction of geometric, probabilistic, or algebraic structures. In particular, the term “subspace” is used here purely in the set-theoretic sense of a subset of $U_{t}$. No linear, metric, or algebraic subspace structure is assumed or required.*

**Remark** **2.** *The canonical parametrization $\iota_{m}=\varphi_{m}^{-1}$ is canonical only in a structural sense (unique once Z is fixed), not in a constructive or algorithmic sense; no claim is made that $\varphi_{m}^{-1}$ is intrinsically accessible from observations.*

**Proof.**  **(i) Injectivity of *ψ*_*m*_.** Since $E_{m}\left(X_{m}\right)=\phi_{m}\left(Z\right)$ with $\phi_{m}$ injective, and $\iota_{m}$ is injective by construction, $\psi_{m}=\iota_{m}\circ \phi_{m}$ is an injective map from Z to $U_{t}$.

**(ii) Isomorphism of the images.** For any m,n, the maps $\phi_{m}:Z\to W_{m}$ and $\phi_{n}:Z\to W_{n}$ are bijections. Thus, $\phi_{n}\circ \phi_{m}^{-1}:W_{m}\to W_{n}$ is a bijection. Composing with $\iota_{m}$ and $\iota_{n}$, we obtain
  $$\Psi_{m\to n}=\psi_{n}\circ \psi_{m}^{-1}:\psi_{m}\left(Z\right)\to \psi_{n}\left(Z\right),$$
  which is therefore a bijection. This establishes the isomorphism of the images.
**(iii) Canonical parametrization.** Since $\phi_{m}$ is a bijection from Z onto $W_{m}$, and Z is already a subset of $U_{t}$, a natural choice is
  $$\iota_{m}=\phi_{m}^{-1}\;(\mathrm{viewed}\;\mathrm{as}\;a\;\mathrm{map}\;\mathrm{into}\;U_{t}).$$

With this choice,

$$\psi_{m}=\iota_{m}\circ \phi_{m}={\mathrm{id}}_{Z},$$

so all $\psi_{m}$ coincide and equal the inclusion of Z into $U_{t}$. Thus, all embedded manifolds share the same image $Z\subseteq U_{t}$. □

**Interpretation** **1.** *The theorem shows that, under minimal assumptions about the generative structure of the data and the behavior of foundational models, all modality-specific embeddings reduce—after an injective embedding into a finite-field shell—to different coordinate parametrizations of the same latent set.*

*(i)*  *Cross-modal alignment is not accidental: all embeddings encode the same latent structure in $U_{t}$.*
*(ii)*  *Transferability is algebraically guaranteed: passing between modalities corresponds to applying a bijection between coordinate charts.*
*(iii)*  *The arithmetic shell is universal:* $U_{t}$ *hosts, simultaneously and compatibly, the encoded representations from all modalities.*

Subsequently, we develop several supplementary results that clarify how the Universal Subspace Theorem (Theorem 1) interfaces with the practice of representation learning. All formal statements follow rigorously from the assumptions established in Section 3 and Section 4. More speculative connections between deep network architecture and the algebraic structure of the FRC are presented as interpretive remarks rather than mathematical propositions.

### 4.1. Representation Lifts and Canonical Embeddings

Theorem 1 shows that for each modality *m* and each observation $x_{m}\in X_{m}$, the learned representation satisfies

$$E_{m}\left(x_{m}\right)=\varphi_{m}\left(z\right),$$

for a unique z∈Z, where $\varphi_{m}:Z\to W_{m}$ is a bijection. Given the canonical choice of embeddings $\iota_{m}=\varphi_{m}^{-1}$, it follows that

$$\iota_{m}\circ \varphi_{m}={\mathrm{id}}_{Z}.$$

This yields the following immediate corollary.

**Corollary** **1** (Canonical Lift)**.** *Let m be any modality and suppose $\iota_{m}$ is chosen as in Theorem 1(iii). Then the map,*

$$L_{m}:X_{m}\to Z\subset U_{t},\;L_{m}:=\varphi_{m}^{-1}\circ E_{m},$$

*satisfies*

$$L_{m}\left(x_{m}\right)=z,\;\mathrm{for}\;\mathrm{the}\;\mathrm{unique}\;z\in Z\;\mathrm{such}\;\mathrm{that}\;E_{m}\left(x_{m}\right)=\varphi_{m}\left(z\right),$$

*for all $x_{m}\in X_{m}$.*

**Proof.**  Immediate from $E_{m}\left(x_{m}\right)=\varphi_{m}\left(z\right)$ and the invertibility of $\varphi_{m}$. □

Thus, each embedding implicitly performs a *latent lift*: it reconstructs the underlying latent state z∈Z up to epistemic limitations of the modality-specific projection. This interpretation is fully consistent with classical sufficiency theory [5,6] and with the empirical role foundational models play in recovering semantic structure [2,24].

### 4.2. Cross-Modal Coherence as Coordinate Change

Theorem 1(ii) implies that for any two modalities m,n, the coordinate representations induced by the embeddings are related by the bijection

$$\Psi_{m\to n}:=\psi_{n}\circ \psi_{m}^{-1}:\psi_{m}\left(Z\right)\to \psi_{n}\left(Z\right).$$

In the canonical parametrization, where $\psi_{m}={\mathrm{id}}_{Z}$ for all modalities, the map $\Psi_{m\to n}$ reduces to the identity on Z.

This yields the following structural result.

**Corollary** **2** (Cross-Modal Consistency)**.** *For the canonical embedding $\psi :Z↪U_{t}$, all modality-specific embeddings satisfy*

$$\psi \circ L_{m}=\psi \circ L_{n}={\mathrm{id}}_{\psi (Z)}.$$

*In particular,*

$$\iota_{m}\left(W_{m}\right)=\iota_{n}\left(W_{n}\right)=\psi \left(Z\right)\subset U_{t}.$$

**Proof.**  Recall from Corollary 1 that for each modality *m* the latent lift is defined by

$$L_{m}:=\varphi_{m}^{-1}\circ E_{m}:X_{m}\to Z.$$

By Definition 2 and the minimal adequacy assumption of Theorem 1, for any observation $x_{m}\in X_{m}$ there exists a unique z∈Z such that

$$E_{m}\left(x_{m}\right)=\varphi_{m}\left(z\right),$$

and therefore

$$L_{m}\left(x_{m}\right)=\varphi_{m}^{-1}\left(E_{m}\left(x_{m}\right)\right)=\varphi_{m}^{-1}\left(\varphi_{m}\left(z\right)\right)=z.$$

Now choose the canonical parametrization from Theorem 1(iii), i.e., select embeddings $\iota_{m}$ such that the composite maps

$$\psi_{m}:=\iota_{m}\circ \varphi_{m}:Z↪U_{t}$$

coincide for all modalities. Denote the common map using ψ. Then, for any two modalities m,n and any observations $x_{m}\in X_{m}$, $x_{n}\in X_{n}$ with

$$E_{m}\left(x_{m}\right)=\varphi_{m}\left(z\right),\;E_{n}\left(x_{n}\right)=\varphi_{n}\left(z\right),$$

we have

$$\psi \circ L_{m}\left(x_{m}\right)=\psi \left(z\right)=\psi \circ L_{n}\left(x_{n}\right).$$

Since ψ is injective on Z, this equality is equivalently

$$\psi \circ L_{m}=\psi \circ L_{n}={\mathrm{id}}_{\psi (Z)}\;(\mathrm{on}\;\mathrm{the}\;\mathrm{image}\;\psi (Z)).$$

Finally, because

$$\psi \left(Z\right)=\psi_{m}\left(Z\right)=\iota_{m}\left(\varphi_{m}\left(Z\right)\right)=\iota_{m}\left(W_{m}\right)$$

for each modality *m*, we conclude that

$$\iota_{m}\left(W_{m}\right)=\iota_{n}\left(W_{n}\right)=\psi \left(Z\right)\subset U_{t},$$

as claimed. □

At a structural level, this explains why foundational models trained on different modalities exhibit coherent semantic alignment when projected into their latent spaces: they are expressing the same latent set in different coordinate systems [7,8,24].

Deep networks alternate linear transformations with nonlinear activations, which is essential for universal approximation [2]. Although no direct algebraic equivalence between nonlinear activations and shell extensions is claimed here, the following conceptual observation clarifies how innovation steps in FRC can be related to expressive steps in deep networks.

**Interpretation** **2** (Nonlinearity as Expressive Expansion)**.** *In the FRC architecture, innovation steps $U_{t}↪U_{t+1}$ introduce new algebraic degrees of freedom. In deep neural networks, nonlinear layers enable an analogous expansion of the set of functions that the model can represent. Thus, both mechanisms serve the role of increasing expressive capacity, but no formal identification is asserted.*

The algebraic complexity of an FRC shell grows with the shell index, which parallels well-known analyses of network expressivity, in which depth increases expressive capacity [11,12,25]. We therefore offer the following additional interpretive connection.

**Interpretation** **3** (Depth and Latent Arithmetic Complexity)**.** *The representational hierarchy generated by alternating linear and nonlinear layers in deep networks mirrors the hierarchical arithmetic complexity of the shell tower $U_{t}\subseteq U_{t+1}\subseteq \cdots$. Both structures exhibit exponential growth in expressive capacity as their hierarchy index increases.*

This is intended as an analogy, not a theorem. Nevertheless, it provides a conceptual lens for understanding why deep networks permit progressively richer abstractions, and why such abstractions align across modalities.

## 5. Discussion

The Universal Subspace Theorem (Theorem 1) shows that, under classical assumptions from multi-view statistical modelling and the algebraic architecture of the Finite Ring Continuum (FRC), all foundational embeddings are coordinate projections of the same latent space Z embedded in the finite-field shell relational symmetry $U_{t}=F_{p}$. This section discusses the broader conceptual implications of this result for representation learning, multimodal alignment, and the emerging theory of foundational models.

### 5.1. FRC as a Structural Explanation for Multimodal Alignment

The empirical observation that independently trained foundational models (linguistic, visual, acoustic, geospatial, etc.) produce embeddings that are not only geometrically meaningful but also mutually compatible has been widely noted in the literature [1,2,3]. However, existing explanations for such alignment are typically heuristic, relying on claims about “shared semantics,” large datasets, or architectural similarity.

The present framework provides a more principled account: the alignment is a necessary consequence of the representation structure, not an empirical accident. Because each foundational embedding $E_{m}$ is minimal adequate for Z and because Z embeds injectively into a single finite-field shell $U_{t}$, the embeddings $\psi_{m}\left(Z\right)$ coincide in a canonical coordinate system. Thus, alignment arises at the level of latent structure rather than learned geometry.

This structural perspective is reminiscent of the role of latent spaces in probabilistic graphical models [7,8] but extends these ideas into an explicitly algebraic setting grounded in the finite-symmetry framework of the FRC [14,15,16]. In this sense, foundational model embeddings are not merely “vector spaces” of features, but coordinate charts on a discrete arithmetic manifold.

Another important empirical illustration of the proposed framework is provided by the grokking phenomenon observed in neural networks trained on small algorithmic datasets [18]. In this setting, models are trained on abstract symbols with no intrinsic attributes, and all structure must be inferred exclusively from relational interactions. Empirically, such models often achieve perfect memorization long before any generalization occurs; generalization then emerges abruptly at a much later stage, accompanied by embedding geometries that recover clear algebraic structure (e.g., cyclic orderings, cosets, and group-theoretic relations).

From the perspective developed here, this behaviour is naturally interpreted as the delayed discovery of a latent relational structure rather than as a gradual statistical interpolation. Optimization dynamics primarily control when a minimal sufficient coordinatization of the latent domain is recovered, but not which structure is ultimately represented. Although the grokking studies do not assume a finite-relational ontology, the observed representational convergence is consistent with—and predicted by—the present framework, in which learned embeddings serve as coordinate charts on an underlying latent relational set once sufficient structure has been resolved.

Furthermore, Corollaries 1 and 2 show that representations learned by foundational models implicitly reconstruct the latent domain Z modulo the observation noise. This echoes the classical dictum that “a sufficient statistic is a lossless representation” [5,6], which is now manifest in modern deep networks. From this vantage point, representation learning can be viewed as the process of discovering an injective parameterization of a latent space that is shared across modalities.

The resulting unification has conceptual consequences:

(i) The functional similarities between representations from different architectures reflect the uniqueness of minimal sufficient statistics, not architectural bias.
(ii) Cross-modal transfer results from the fact that all representations factor through the same latent domain Z.
(iii) Latent reconstruction occurs even without supervision, provided the training objective incentivizes sufficiency-like behavior (e.g., contrastive learning, masked prediction).

Thus, sufficiency rather than optimization heuristics appears as the proper lens for understanding the universality of modern representation learning: it resolves the memorization-generalization tension highlighted by the ability of over-parameterized networks to fit arbitrary labels [17], provides a structural reading of abrupt emergent abilities observed at scale [18,19], explains the selectivity of transfer scaling through latent overlap rather than compute alone [20], and clarifies why knowledge can be present in learned representations yet remain non-elicitable without suitable coordinate alignment or probing [21].

Despite its unifying explanatory power, the proposed framework also introduces several potential risks and limitations that merit careful consideration. First, by emphasizing latent adequacy and structural inevitability, the framework may underplay the practical role of optimization dynamics, architectural inductive biases, and training curricula in determining convergence speed, stability, and accessibility of adequate representations; while these factors do not create latent structure, they strongly influence whether and when such structure is recovered in practice.

Second, the identification of generalization with latent reconstruction risks oversimplifying settings in which data-generating processes are non-stationary, adversarial, or only weakly factorizable through a shared latent domain, where no stable or minimal adequate representation may exist.

Finally, the framework’s interpretability hinges on the existence of well-defined coordinate changes between representations; in high-dimensional models, such coordinate alignment may be computationally intractable or empirically opaque, limiting the framework’s immediate operational utility for model analysis or control. These considerations do not invalidate the proposed perspective, but they highlight the need for caution in its application and for further work clarifying the boundary between latent structural explanations and the contingent effects of learning dynamics and data collection.

### 5.2. FRC Shells as Universal Host Spaces

The embedding of all learned representations into the same FRC shell $U_{t}$ suggests that foundational models inhabit a universal representational domain determined by discrete arithmetic structure. The fact that $U_{t}$ is finite yet supports rich internal symmetry parallels recent arguments that large-scale models implicitly operate in low-dimensional but highly structured latent spaces [9,10].

The FRC framework strengthens and algebraically grounds this viewpoint:

(i) The latent space is not assumed to be Euclidean or continuous, but finite, relational, and symmetry-complete.
(ii) Distinct modalities embed into the same shell, implying a common underlying geometry.
(iii) The shell hierarchy $U_{t}\subseteq U_{t+1}\subseteq \cdots$ provides a natural progression of expressive capacity, paralleling the depth hierarchy in neural networks [11,12].

This suggests an intriguing interpretation: foundational models partake in a discrete analogue of geometric unification, expressing diverse data domains within a common algebraic manifold.

### 5.3. Connections to Theories of Inductive Bias and Universality

The FRC perspective complements existing theoretical accounts of deep learning inductive bias, such as hierarchical compositionality [25], group symmetry and invariance [9], and universal approximation [26]. In particular:

(i) The minimal sufficiency framework explains why learned embeddings tend toward canonical forms.
(ii) The finite-field shell structure provides a potential candidate for the “universal latent model” implicitly assumed in multimodal learning.
(iii) The discrete nature of $U_{t}$ aligns with emerging perspectives that large-scale models behave as information compressors rather than continuous function approximators [13].

The algebraic viewpoint therefore enriches, rather than replaces, existing foundations for the theory of deep learning.

### 5.4. Representational Convergence Across Biological and Artificial Learners

The relational and finite-informational ontology underlying the FRC framework invites a broader interpretation regarding the nature of learned representations in both artificial and biological cognitive systems. If the latent domain Z is fundamentally finite, attribute-free, and embedded within a symmetry-complete arithmetic shell $U_{t}$, then any agent attempting to infer structure from the external world must extract relational information from the same underlying finite substrate. According to this view, representational alignment between independently trained artificial systems is not accidental, but a structural consequence of recovering compatible coordinate charts of a single latent shell.

A cautious extension of this perspective applies to biological cognition. Modern theories of neural representation—including predictive coding, efficient coding, and manifold learning [27,28,29]—suggest that the brain constructs internal relational models of the world that are modality-invariant and compressed. If the external world is fundamentally finite and relational, as posited by the FRC ontology, then the internal representations acquired by biological learners may likewise be understood as coordinate embeddings of subregions of the same latent domain Z.

This interpretation does not claim that biological and artificial learners share mechanistic similarity, nor does it suggest that neural computation implements finite-field arithmetic in any literal sense. Rather, the claim is representational: if all observers operate within a finite relational universe and learning seeks relational sufficiency, then diverse learners—animals, humans, and modern machine learning systems—may converge toward structurally compatible internal representations despite differences in architecture, modality, or training history.

### 5.5. Implications for Artificial General Intelligence

Within this interpretive framework, it is possible to articulate a bounded and conceptually grounded statement about the nature of artificial general intelligence (AGI). If intelligence is understood not as a specific algorithmic mechanism, but as the capacity to infer, compress, and manipulate relational structure within a finite latent universe, then the FRC ontology implies that any sufficiently general learner must approximate representations that are coordinate charts on $Z\subseteq U_{t}$. In this sense, general intelligence—biological or artificial—corresponds to the ability to recover task-relevant relational invariants of the latent shell, rather than to emulate a particular biological process.

This perspective differs from anthropomorphic or mechanistic definitions of AGI. It does not assert that artificial systems replicate cognition, nor that human intelligence is reducible to machine computation. Instead, it highlights a structural convergence: if both systems are extracting relational information from the same finite universe, then even radically different learning mechanisms may converge to compatible internal representations. The empirical success of foundational models on tasks involving human-like semantic or conceptual reasoning [4,30] may therefore reflect a shared relational target rather than a convergence of implementation.

Seen in this light, AGI becomes a question not of copying the human mind, but of constructing learners capable of efficiently navigating the relational geometry of a finite universe. The FRC framework suggests that such navigation is fundamentally possible, and indeed naturally arises in systems that acquire minimal adequate relational representations from their observational data. Future work may explore how learning architectures can more directly leverage finite-field structure, and whether biological and artificial systems exhibit deeper commonalities in how they partition and operate on the latent shell.

## 6. Finite Ring Geometry of Foundational Embeddings

### 6.1. Empirical Observations Across Modalities

A consistent and widely documented empirical phenomenon in modern foundational models is that their learned embeddings concentrate on the surface of a high-dimensional hypersphere. This behavior is observed across modalities—vision, language, audio, and multimodal systems—and arises both as an implicit consequence of high-dimensional geometry and as an explicit design choice in state-of-the-art representation-learning methods.

In computer vision, contrastive frameworks such as SimCLR [31], MoCo [32], and CPC [33] explicitly normalize embeddings to unit $\ell_{2}$-norm, forcing representations to lie on a hypersphere. Large-scale multimodal systems such as CLIP [4] apply the same normalization to both image and text embeddings, enabling aligned semantic structure across modalities.

In natural language processing, contrastive text encoders such as SimCSE [34] produce sentence embeddings that are likewise constrained to the unit sphere. Moreover, contextual word vector analyses show that embeddings from large transformers (e.g., BERT, GPT-2) concentrate on a thin hyperspherical shell [35], indicating that hyperspherical geometry emerges even without explicit normalization.

These findings are reinforced by theoretical analyses such as that by [36], which demonstrate that contrastive objectives promote *uniformity* on the hypersphere; and by classical high-dimensional geometry, where concentration-of-measure phenomena naturally place high-dimensional vectors near the sphere [37,38].

From the perspective of the Finite Ring Continuum (FRC), this empirical geometry is not incidental. In the FRC ontology, the latent structure of the universe is modelled as a finite, symmetric, attribute-free set of primitive elements embedded in an arithmetic shell $U_{t}$. This shell carries a uniform relational geometry with no preferred scale or distinguished radius; when embedded into a Euclidean vector space, such a finite uniform domain admits only one geometrically unbiased representation: a hypersphere. That is,

$$Z\subseteq U_{t}\;\;⟹\;\;\mathrm{Emb}\left(Z\right)\subseteq S^{d-1},$$

for any Euclidean embedding $\mathrm{Emb}:U_{t}↪R^{d}$ that preserves relational symmetries.

The hypersphere, therefore, is the continuous shadow of a finite arithmetic shell: uniform radial magnitude corresponds to the absence of intrinsic attributes, and direction encodes relational information. The collapse of empirical embeddings onto a thin hyperspherical manifold is thus precisely the structure expected from a finite, relational latent universe. Foundational models appear to reconstruct, through training dynamics rather than explicit design, the geometric signature of finite-shell arithmetic predicted by FRC.

The prevalence of hyperspherical embeddings provides independent support for the central thesis of this work. If latent structure is fundamentally finite and relational, then any representation mechanism that attempts to recover this structure from partial observations must produce embeddings compatible with the symmetries of $U_{t}$. The observed hyperspherical behavior of modern foundational models therefore aligns naturally with the FRC interpretation: embeddings are coordinate charts on a finite relational shell, and the sphere in Euclidean space is the unique continuous manifold that preserves this relational symmetry.

### 6.2. Quantization, Hypersphere Radius, and Representational Capacity

Although embeddings produced by foundational models are often described in continuous terms, real-world computation operates exclusively on finite, quantized numerical representations. Floating-point formats implement a discrete subset of R, determined by fixed mantissa and exponent precision [39,40]. Consequently, any embedding vector

$$x=\left(x_{1},\ldots ,x_{d}\right)\in R^{d}$$

generated by a digital model is, in practice, an element of a finite Cartesian product of quantized sets:

$$x_{i}\in Q\subset R,\;\left|Q\right|<\infty .$$

When embeddings are normalized to lie on a hypersphere [31,32,34,35,36], this quantization acquires a direct geometric interpretation. For a fixed quantization step Δ, the number of distinguishable points on a *d*-dimensional sphere of radius *R* scales approximately like

$$N\left(R\right)∼C_{d}{\left(\frac{R}{\Delta }\right)}^{d-1},$$

where $C_{d}$ is a dimension-dependent constant reflecting spherical packing bounds [41]. Thus, for fixed embedding dimension and resolution, the representational capacity grows with the radius of the hypersphere.

In the context of the Finite Ring Continuum (FRC), this is precisely the expected behavior. A finite relational latent domain Z admits only finitely many distinguishable representational states. When embedded into a Euclidean space for computation, these states must occupy a finite set of approximately uniformly spaced points on a hypersphere. The radius *R* then reflects the effective size of the latent shell being represented under a fixed quantization scheme. This correspondence between discrete hyperspherical geometry and finite relational structure provides further support for interpreting embedding spaces as finite-shell projections rather than continuous manifolds.

### 6.3. Gödel Encoding and Collapse into a Single Finite Ring

Quantized hyperspherical embeddings also admit a remarkable algebraic property: all coordinate dimensions can be encoded into a *single* element of a finite ring without loss of information. This follows from classical Gödel-style encodings [42,43], where finite tuples of integers are mapped injectively into a single integer using prime-power factorizations.

It should be noted that Gödel-style encodings are invoked here only to establish injective representability. They do not preserve geometric or learning-related structure. Their relevance in the present context is that, within the Finite Ring Continuum, prime finite fields provide a canonical algebraic scaffold in which such injective encodings are non-arbitrary and compositional, even though geometry and optimization structure are not preserved.

Let $x=(x_{1},\ldots ,x_{d})$ be a quantized embedding vector, where each $x_{i}$ is an integer in a bounded range. Selecting distinct primes $p_{1},\ldots ,p_{d}$, one may define the Gödel map

$$G\left(x\right):=p_{1}^{x_{1}}p_{2}^{x_{2}}\cdots p_{d}^{x_{d}}.$$

Because the fundamental theorem of arithmetic guarantees unique factorization, the map *G* is injective on the finite domain of representable embeddings. Reducing G(x) modulo a sufficiently large prime *q* yields

$$\bar{G}\left(x\right):=G\left(x\right)\;\mathrm{mod}\;q\in F_{q},$$

and as long as the modulus *q* exceeds the maximum value attained by G(x) on the relevant embedding set, no collisions occur.

Thus, any finite collection of embedding vectors—even if treated as points on a continuous hypersphere—can be represented as distinct elements of a *single* finite field $F_{q}$. In the FRC interpretation, this illustrates that the apparent multi-dimensional structure of embedding spaces is a representational artifact rather than a fundamental geometric property. The information content of an entire *d*-dimensional spherical manifold can be collapsed into a subspace of a single arithmetic shell $U_{t}$, with all coordinate dimensions encoded relationally.

This explicit constructive mapping reinforces the central thesis of the FRC framework: multi-dimensional continuous embeddings are coordinate expressions of finite relational structure, and all observable complexity can be understood as arising from arithmetic relations within a single finite universe. We would like to note, however, that Gödel encodings serve only to demonstrate representational equivalence within a finite ring. They are not proposed as computational mechanisms for gradient-based training.

## 7. Conclusions

This work has introduced a unified mathematical framework that connects representation learning in modern foundational models with the algebraic architecture of the Finite Ring Continuum (FRC) [14,15,16]. Building on classical principles of minimal sufficiency [5,6] and multi-view latent variable modelling [7,8], we demonstrated that foundational embeddings from arbitrary modalities—textual, visual, acoustic, geospatial, or otherwise—can be interpreted as coordinate embeddings of a single latent set Z inside a shared finite-field arithmetic shell $U_{t}$. Once again, this article does not derive FRC from neural optimization. Instead, it shows that the representational geometry emerging in foundational models is consistent with, and predicted by, the FRC ontology.

The Universal Subspace Theorem (Theorem 1) constitutes the central theoretical result of the paper. It shows that, under minimal and well-justified assumptions, all learned representations factor through bijective images of the same latent domain. In a canonical parametrization, these embeddings coincide exactly. This provides a rigorous explanation for the empirical phenomenon of cross-modal alignment observed across large-scale deep learning systems [1,2,3]: alignment emerges not as a consequence of architectural similarity or shared training objectives, but as a structural property induced by the existence of a common latent world variable.

Beyond this, we have shown that representation learning implicitly reconstructs the latent state *z* up to bijection; that cross-modal consistency follows naturally from the uniqueness of minimal sufficient statistics; and that the finite-field shell $U_{t}$ serves as a universal host space for learned representations. The resulting perspective suggests a view of foundational models not as collections of modality-specific encoders, but as coordinate charts on a discrete arithmetic manifold shared across all modalities.

The interpretive results of Section 4.1 and Section 4.2 indicate further connections between deep learning and FRC. While no formal equivalence between nonlinear network operations and FRC innovation steps is claimed, the parallel between network depth and shell hierarchy offers a promising avenue for future theoretical development [11,12]. More broadly, the discrete and relational structure of the FRC aligns with emerging perspectives that emphasize the role of compression, abstraction, and latent geometry in large-scale learning systems [9,13].

Taken together, these findings motivate several directions for future work: (i) axiomatizing learning objectives as approximations to minimal adequacy, (ii) developing explicit algorithms for learning projection maps $g_{m}:Z\to X_{m}$, (iii) relating neural network depth more formally to arithmetic shell complexity, and (iv) extending the theory toward a general algebraic account of multimodal learning and foundational model universality.

While the directions outlined above are structurally motivated by the FRC framework, several challenges remain. First, extending results from symmetry-complete key-frame shells to fully general composite cardinalities requires careful control of coupled subshell structure and bookkeeping across Chinese-remainder components. Second, establishing systematic correspondences with continuum formalisms demands precise statements of approximation regimes and observer-horizon limits. Third, translating algebraic causality and finite dynamics into testable physical models involves separating structural predictions from scale-dependent effects.

However, these difficulties are not conceptual inconsistencies of the framework, but technical and methodological challenges inherent in connecting a finite relational foundation with established infinite or continuum-based theories.

By grounding representation learning in the finite, relational, and symmetry-complete structure of the FRC, this work contributes to a deeper and more principled understanding of why foundational models exhibit such remarkable generality, and how their latent spaces may ultimately reflect a shared underlying arithmetic structure across all modalities of human and machine perception.

## Data Availability

The original contributions presented in this study are included in the article. Further inquiries can be directed to the corresponding author.
